# Serological Susceptibility to Measles Among International Students in South Korea After a Cluster of Cases: A Cross-Sectional Study

**DOI:** 10.3390/tropicalmed11060150

**Published:** 2026-05-30

**Authors:** Minyu Qin, Na Young Hong, Shin Woo Kim, Ji Hyuk Park, Seok Ju Yoo, Sung Jun Park, Younjoo Kim, Sang Yun Cho, Sook Hee Park, Hyun Jun Kang, Byeong Ryeon Kim, Mina Lee, Hyejin Hong, Minjei Lee, Myung Jae Hwang, Sookhyun Kim, Myung Hee Kim, Min A. Lim, Youkyoung Kim, Kwan Lee

**Affiliations:** 1 Department of Preventive Medicine, College of Medicine, Dongguk University, Gyeongju 38066, Republic of Korea; qmydgu77@gmail.com (M.Q.);; 2 Gyeongbuk Center for Infectious Diseases Control and Prevention, Andong 36759, Republic of Korea; 3 Department of Internal Medicine, School of Medicine, Kyungpook National University, 130, Dongdeok-ro, Jung-gu, Daegu 41944, Republic of Korea; 4 Daegu Center for Infectious Diseases Control and Prevention, Daegu 41940, Republic of Korea; 5 Division of Infectious Disease Response, Gyeongbuk Regional Center for Disease Control and Prevention, Korea Disease Control and Prevention Agency, Daegu 41914, Republic of Korea; 6 Division of Public Medicine, Gyeongsangbuk Do Provincial Government, Andong 36759, Republic of Korea

**Keywords:** measles, immunoglobulin G, seroepidemiologic studies, vaccination

## Abstract

Background: Even though South Korea had eliminated measles in 2006, a localized cluster of measles occurred in a university dormitory in Gyeongsangbuk-do, South Korea, in 2024, involving 22 international students. This study aims to explore measles transmission and to inform future preventive measures. Methods: In a cross-sectional study, 299 international students from two local universities underwent a self-reported questionnaire survey and targeted serological testing by enzyme-linked immunosorbent assay two months after the cluster of measles cases. Statistical analysis used Pearson’s chi-square test, Fisher’s exact test, multivariate logistic regression, and Firth’s penalized logistic regression. Results: The overall seropositivity was 79.6%, and 78.0% among participants aged ≤30 years. Among multivariate analyses, nationality was independently associated with seropositivity (aOR up to 8.35 for Chinese students). Conclusions: These findings underscore the immunity gaps among international students, with seropositivity remaining below the 95% threshold required for herd immunity. Targeted serological screening and catch-up vaccination may help to improve immunity in mobile populations.

## 1. Introduction

Measles is one of the most contagious viral diseases, with transmission occurring from four days before to four days after rash onset. Unvaccinated individuals are at higher risk of measles infection and may face more severe complications, compared to those vaccinated. Unvaccinated persons comprise the majority of fatality cases worldwide [1], especially among children under five years of age. According to the reported data from the Centers for Disease Control and Prevention, between one and three out of every 1000 children infected with measles are at risk of death due to neurological and respiratory issues [2]. The South Korean historical vaccination policy suggests presenting proof of two vaccine doses among preschool children and conducting large-scale supplementary immunization activities (SIAs) for adolescents aged 8 to 16. This strategy elevated vaccination coverage rates of preschool children and adolescents to 99% and 97%, respectively. In 2006, the South Korean government declared measles elimination, following the 5-year measles elimination program [3]. During the coronavirus disease (COVID-19) pandemic, routine immunization programs were disrupted globally, resulting in immunity gaps among younger cohorts [4,5,6,7,8]. While quarantine measures and reduced international travel served to prevent disease transmission among countries during the COVID-19 pandemic, they also disrupted routine immunization services worldwide. In the early stage of the pandemic, about 170 countries and territories globally reported a decline in the number of administered doses of the first dose of measles-containing vaccine (MCV1) [5]. This disruption and stagnation of routine immunization programs may have contributed to the increased risk of measles transmission in the post-pandemic period.

International students in South Korea are generally subject to university admission and visa-related health requirements; the most common is a tuberculosis certificate. In contrast, measles vaccination or measles–mumps–rubella vaccination certification is introduced in some universities’ institutional policies requiring students applying for dormitory residence [9,10] after the 2024 measles cluster, rather than a nationwide mandatory requirement. Meanwhile, the implementation of local policies may be influenced by institutions, timing, and residence policy, indicating a potential gap between national measles elimination goals and campus-level preventive measures.

South Korea may face the risk of a measles cluster, although it maintained a high vaccination coverage of over 97% during the COVID-19 pandemic (2020–2023). Measles cases continue to be reported according to the data from the South Korean Centers for Disease Control: 6 in 2020, 8 imported cases in 2023, and 49 imported and import-associated cases in 2024. The number of 78 confirmed measles cases was reported in 2025, which is significantly higher than in 2024. The previous evidence suggests that susceptibility among young adults is also notable [11,12,13]. The prevalence of childbearing women aged 20–24 increased significantly from 9.8% in 2013–2015 to 25.0% in 2021–2024 [13]. Additionally, the investigations on measles antibody seroprevalence among healthcare workers in South Korea found significant variation in age groups [14], particularly in cohorts born after 1995 [15]. A similar age-related reduction in seroprevalence was also shown in a survey of South Korean immigrants [16]. These gaps are particularly noticeable in highly mobile populations [17,18,19], such as international students. In April 2024, a measles cluster in a university dormitory in Gyeongsangbuk-do involved 22 international students [20]. This event highlights potential immunity gaps in highly mobile populations. This may be partly associated with heterogeneous vaccination backgrounds, as these populations often reside in densely populated settings and may have incomplete vaccination histories.

Advanced evidence suggests that measles transmission dynamics may be associated with population mobility, heterogeneous immunity, clustered susceptibility, migration, and social contact patterns, even in high-vaccine-coverage populations [21,22,23]. A gathering of susceptible individuals may increase the risk of a measles cluster by around 20% [23]. International students represent a particularly vulnerable population with the relevant characteristics frequent cross-border mobility, different vaccination backgrounds, and clustered living. Therefore, although the publicly available surveillance summaries do not classify by domestic students, international students, or non-student status in national annual measles case counts, understanding measles immunity and susceptibility among international students remains important for public health preparedness and infection prevention.

This study aimed to offer data for better health management and vaccination records and to provide evidence-based recommendations to strengthen immunization policies for supporting measles control efforts in the campus context.

## 2. Materials and Methods

### 2.1. Study Design and Data Collection

A cross-sectional seroepidemiological study was conducted among international students enrolled at two universities in Gyeongsangbuk-do, South Korea, from October to November 2024. The serological testing and questionnaire were conducted two months after the cluster of measles cases occurred in the study.

Participants were selected using stratified random sampling based on nationality and the proportional distribution of international students enrolled at the targeted universities. Based on the Ministry of Education’s statistics on international students in South Korea and Gyeongbuk by country (quoted on 1 April 2023), selected students were mainly from China, Vietnam, and Central Asia (Uzbekistan and Kazakhstan), constituting over 75% of all international students. A total of 300 participants were recruited, including 130 Chinese, 75 Vietnamese, and 95 Central Asian students. These national groups were selected based on both proportional representation and epidemiological relevance to measles importation risk.

The inclusion criteria were enrollment as an international student at one of the target universities and provision of informed consent. Exclusion criteria were: (1) a diagnosed condition known to compromise immunity, (2) undergoing regular medical treatment, or (3) declining to participate. The sample size was estimated using G*Power software (version 3.1.9.7, Heinrich Heine University Düsseldorf, Düsseldorf, Germany) based on an expected measles seroprevalence of 50%, a 95% confidence level, and a maximum allowable error of ±10%. A prevalence of 50% was conservatively assumed to maximize sample size estimation. Stratified random sampling by nationality was subsequently applied to ensure representation of the major international student groups entering Gyeongsangbuk-do. A total of 299 international students were finally included in the study sample, with one Chinese student excluded due to a disease affecting immunity.

### 2.2. Data Collection

Participants were invited by email and gathered in a classroom to finish the self-reporting pre-screening questionnaire using a pencil and paper after obtaining their consent. Researchers from the Centers for Disease Control and Prevention and Dongguk University answered questions and conducted questionnaire collection on-site. The questionnaire included the participant information sheet and consent form, which were translated into English, Chinese, Vietnamese, and Uzbek (which also served Russian-speaking participants). To ensure linguistic accuracy and cultural appropriateness, native-speaking translators from the relevant countries were engaged in the translation and verification process, with real-time clarification regarding the study content. Demographic and background data (age, gender, health status, vaccination history, and overseas travel experience) were collected through a standardized questionnaire adapted from the WHO Vaccination Coverage Cluster Survey Reference Manual. Measles and vaccination history were self-reported and could not be verified using official immunization documentation.

### 2.3. Serological Testing

Measles-specific IgG antibody levels were quantified using a commercially available Enzyme-linked immunosorbent assay (ELISA) kit (Measles IgG ELISA, Bio-Rad, Marnes-la-Coquette, France), which is a well-established method for assessing immune status and identifying seronegative individuals for potential vaccination [19]. Clinical pathologists gathered the blood samples, which were sent to the Seegene company for ELISA testing. The procedure followed the manufacturer’s instructions. Briefly, serum samples were diluted 1:10 in wash buffer (100 mL + 900 mL D/W). Subsequently, a further dilution to 1:1000 was performed using the provided diluent. Each 100 µL of sample was dispensed into the 96 wells of the ELISA kit according to the conditions of positive, negative, or cut-off control. Samples were incubated at 37 °C for 45 min, and then 100 µL of antibody conjugate was added to the wells and incubated for 45 min at 37 °C. An equal volume of 3,3′,5,5′-tetramethylbenzidine (TMB) substrate solution was added to each well, followed by a 15 min incubation in a green room (or dark room), to allow for color development. The enzymatic reaction was terminated by adding 100 µL of stop solution to each well. Absorbance was read at 450 nm with 620 nm as the control wavelength. According to the manufacturer’s protocol, measles-specific IgG levels were interpreted as negative (<0.80), equivocal (0.80–1.19), and positive (≥1.20). Samples with equivocal results were retested to confirm classification.

### 2.4. Ethical Considerations

Ethical approval was granted by the Institutional Review Board of Dongguk University Gyeongju Hospital (IRB No: 110757-202409-HR-01-03). Written informed consent was obtained from all participants before enrollment.

### 2.5. Statistical Analysis

Missing or ‘Unknown’ responses were not imputed. For descriptive and bivariate analyses, available-case analysis was used, and the denominator for each variable was reported where applicable. For multivariable regression, participants with missing values for variables included in the model were excluded from the corresponding model. In addition to studies conducted on the entire research population, a restricted analysis was performed on participants aged 17 to 30 years to reduce the influence of older participants, who may have different natural exposure histories, undocumented infections, or prior vaccination. This age restriction was intended to reduce heterogeneity in age-dependent immunity patterns and improve comparability within the predominantly young international student population, rather than to define a universal birth cohort across countries. Descriptive statistics were used to summarize participants’ characteristics and IgG seropositivity. Pearson’s chi-square test, Fisher’s exact test, and Kruskal–Wallis chi-square test were applied to find potential associations between categorical variables. Variance inflation factors (VIFs) were used to estimate multicollinearity among candidate variables. A VIF greater than 5 was considered indicative of potential multicollinearity. Variables were selected for multivariable models based on epidemiological relevance, the prior literature, and statistical association in bivariate analyses. Variables with *p* < 0.20 in bivariate analysis or those considered a priori important were entered into the initial model. Baseline variables that were considered relevant to public health, that were covariates, or showed a univariate relationship with the outcome were respectively entered into the Firth’s penalized logistic regression and multivariate logistic regression models for different age groups, including gender, age, nationality, education level, and history of overseas travel. Firth’s penalized logistic regression was applied to reduce small-sample bias and address potential separation issues. A *p*-value < 0.05 was considered statistically significant. All the analyses and visualizations were conducted by IBM SPSS Statistics version 21 (IBM Corp., Armonk, NY, USA) and the R project (version 4.5.2).

## 3. Results

The questionnaire was distributed to 300 participants in total. There was a 100% response rate. A final analytical sample of 299 individuals was obtained after one participant was eliminated because of a pre-existing medical condition. The baseline characteristics of the cohort, obtained from self-reports, are summarized in Table 1. The sample comprised 173 (57.9%) males and 126 (42.1%) females, with the majority falling within the 21–30 age group. In terms of nationality, 43.1% of the participants were from China, 25.1% from Vietnam, and 31.8% from Central Asia. Notably, only 25.1% self-reported prior measles vaccination, with a substantial proportion (22.4%) being uncertain of their status. The self-reporting results may indicate the participants’ comprehension of their measles vaccination status, but they do not accurately reflect the measles IgG situations.

The overall seroprevalence of measles IgG antibodies was 79.6% (95% CI: 74.7–83.9). As detailed in Table 2, seroprevalence differed significantly across key demographic strata, particularly in nationalities. Variation was also observed in terms of age, with rates increasing from 70.2% in the 17–20 age groups to 84.0% in the 21–30 group, and reaching 100% among participants aged 31 years or older (*p* = 0.001). Furthermore, female participants exhibited a significantly higher seroprevalence (88.1%) compared to males (*p* = 0.002). The academic program was also a significant factor, with graduate students showing the highest immunity compared to undergraduates and language institute students (*p* < 0.001). Participants under 31 years old showed similar significant differences in genders, age groups, nationalities, and educational attainments. This finding suggested a statistically significant association between these characteristics and seroprevalence.

**Figure 1:**
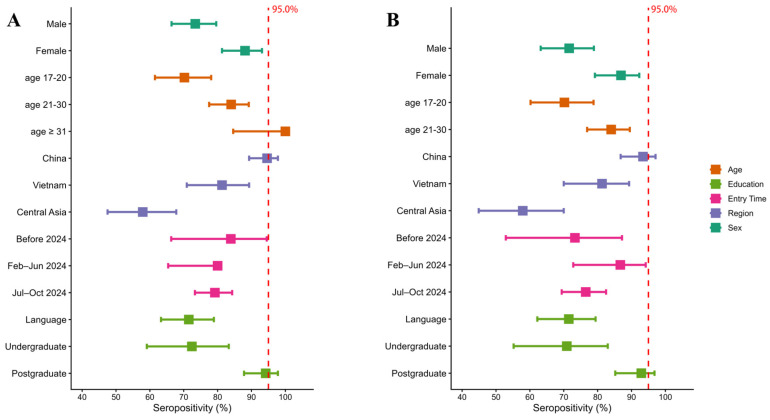
Forest plot of measles IgG seropositivity by general characteristics. (**A**): Seropositivity measured among all age groups (*n* = 299); (**B**): seropositivity measured among participants aged ≤30 years (*n* = 277).

As illustrated in Figure 1, the majority of subgroup-specific seropositivity was below the 95% threshold (WHO recommendation) in both panel A and panel B. This level was only reached by the over-30 age group. Additionally, seropositivity of participants from China or students in post-graduate research was close to the 95% threshold among all age groups (panel A). The same result was found among participants without age bias as well (shown in panel B).

We further examined the association between seropositivity and various health-related factors, as shown in Table 3. It was demonstrated in both the full age group and the group of participants aged 30 and below that no statistically significant associations were found with self-reported vaccination status, number of vaccine doses, history of measles infection, current health condition, or type of residence. In contrast, a history of overseas travel within the past year was strongly associated with higher seropositivity (93.8% vs. 72.5%, *p* < 0.001).

**Figure 2:**
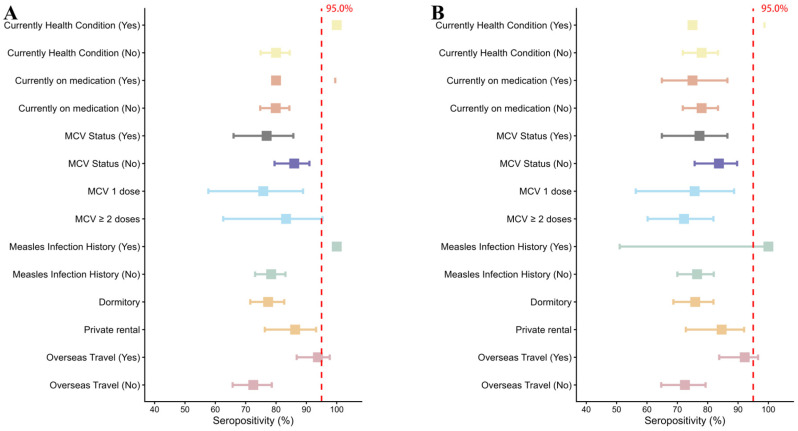
Forest plot of measles IgG seropositivity by self-reported health status. (**A**): Seropositivity was measured among all age groups (*n* = 299); (**B**): seropositivity was measured among participants aged ≤30 years (*n* = 277). MCV: measles-containing vaccine.

Participants who reported having traveled abroad or having a history of measles were close to the 95% threshold in the entire population (Figure 2A). The other subgroups remained below the cutoff, covering all vaccination-related categories.

The multivariate logistic regression results shown in Table 4 revealed that nationality continued to be a single, independently associated factor of measles IgG result. The odds of seropositivity were significantly greater in international students from China (adjusted OR = 8.35, 95%CI: 2.67–28.50) and Vietnam (adjusted OR = 2.43, 95%CI: 1.07–5.77) than in those from Central Asia. Gender, age group, academic level, or international travel history were no longer substantially correlated with seropositivity, in contrast to the univariate analysis results (all *p* > 0.05). This suggests that gender or other model-related factors may mediate or confound the impact.

**Figure 3:**
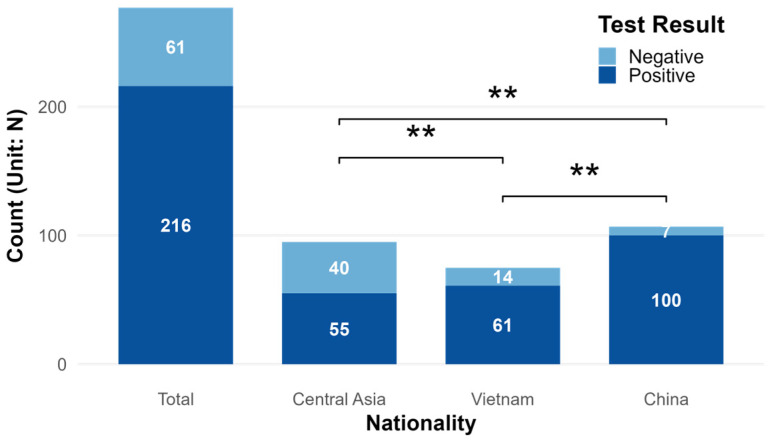
Measles IgG seropositivity by participant nations (*n* = 277). Statistical analysis: Fisher’s exact test. **: *p* < 0.001.

The distribution of serostatus varies clearly between nations (shown in Figure 3). The greatest number of seropositive individuals was seen in China, followed by Vietnam and Central Asia. The prevalence of seropositive and seronegative groups was evenly distributed in Central Asia. These results support the multivariable analysis finding that significant heterogeneity exists among nations.

## 4. Discussion

Measles vaccination using live-attenuated virus strains produces strong humoral protection, as evidenced by the high persistence of specific IgG for several years. The antibody titer can remain above the protective threshold for more than 14 years [24]. As a result, the high seroprevalence result indicates a strong vaccine-induced immunity wall. Our study’s inadequate overall measles antibodies seroprevalence of 79.6% among international students is worryingly below the WHO-recommended threshold of 95%, suggesting the vulnerability of international students. This could point to possible immunity gaps that need to be addressed in campus contexts.

The disparities in seropositivity by nationality were observed in our study. Vietnamese and Chinese students showed 2- to 8-fold higher odds of seropositivity compared with Central Asian students, which may reflect differences in immunization programs and epidemiological histories in their countries of origin. The high seropositivity of Chinese students may be a result of the implementation of the national Expanded Program on Immunization (EPI) since the 1980s [25], and the nationwide Supplementary Immunization Activity (SIA) in 2010 [26]. Furthermore, the measles-containing vaccine first-dose (MCV1) (measles–rubella combined vaccine) and measles-containing vaccine second-dose (MCV2) (measles–mumps–rubella combined vaccine) were administered at the 8th month and 18th month in China. The government traditionally conducts large-scale SIA targeting preschool children with the aim of high immunization coverage rates. The Vietnamese government recommended two-dose measles vaccination at the 9th month and 18th month, respectively. An SIA was also carried out to cope with the epidemic in 2024, with a remarkable result in terms of an increase in vaccine coverage [27]. Paradoxically, despite the high seropositivity, self-reported vaccination was low among Chinese students, likely due to recall bias or incomplete records. Conversely, the difference in Central Asia may reflect differences in immunization history, documentation, access, or outbreak exposure regarding these “campaign-style” immunization programs that successfully increase the target population’s seroprevalence. Variations in vaccination history, documentation systems, and access to immunization services may be the cause of the reported regional variances in seroprevalence. According to WHO estimates, MCV2 coverage in Central Asia was 96% in 2024, with an incidence rate of 1022.56 cases per million in 2024 (the average of provisional data of MCV2 coverage and incidence in Uzbekistan and Kyrgyzstan based on monthly data reported to WHO as of December 2025 was used to reflect measles vaccination coverage and incidence throughout Central Asia) [28]. However, 57.9% seropositivity among Central Asia students was found in our study, with 48.9% self-reporting a vaccine history. Notably, no significant association between self-reported vaccine history and seropositivity was observed. Self-reported data are inherently biased, especially in young adult populations who had immunizations as children and may not have an accurate memory of their immunization history. Thus, self-reported vaccination status should be regarded with caution as an indicator of immunological protection. On the other hand, serological testing can provide a more objective measure of population immunity. This discordance highlights a broader methodological challenge in sero-epidemiological studies that behavioral or self-reported indicators may not accurately reflect biological immunity. The promotion of vaccination documentation systems and serological screenings, or incorporating validated immunization records into health assessment procedures, especially for mobile and high-risk populations, is therefore significantly important.

Because of the protection from the universal vaccination program introduced in the 1980s, the measles burden suffered by children has reduced, while adults are currently the most susceptible group in South Korea. We had to consider the association between seropositivity and nature infection 30 years after two-dose vaccination, given the increasing trend in the potentially susceptible population 20 years after the second dose, and the possible seronegative population after 30 years [11]. This is the reason why the data from participants aged 17–30 years were separately subjected to a statistical analysis in our study. Additionally, some research supported that younger adults have a lower measles seroprevalence. In the absence of natural boosting due to low disease circulation, the younger generation born during the period of established vaccination programs may constitute a cohort with vaccine-induced immunity [29]. However, vaccinated individuals may become more susceptible to measles after 10 to 20 years, perhaps as a result of decreasing antibody levels over time [30,31,32,33]. Participants under 30 years old in our study who were born in the 95 cohort have a seroprevalence of almost 78.5%. However, we did not find any significant variations between age groups. The relatively small sample size and sample imbalance can be attributed to this result.

We observed no significant variation in gender in this cohort. This is different from the findings showing females had significantly higher IgG titers at the same age [34] due to the stronger humoral and cell-mediated responses post-vaccination, potentially influenced by hormonal and genetic factors [35,36]. The disparity can be explained by the presence of confounders such as age and nationality, as well as short-term exposure risk patterns, which may obscure the long-term immunological differences.

Our study provides initial seroepidemiological evidence of measles immunity gaps among international students in Central Asia—a growing yet often overlooked population. The combination of high-density living, frequent international travel, and inadequate immunity presents challenges in infectious disease control [37,38]. Given global mobility, maintaining sufficient population immunity is still crucial to measles control. Previous studies have suggested that nearly 100% of measles cases in areas with interrupted endemic transmission (and vaccine coverage > 95%) are travel-related [39,40]. Inadequate case management, delayed measles case reporting, or a lack of measles awareness among healthcare workers may potentially contribute to the spread of measles [41]. Our univariate analysis found that participants with recent foreign travel history had higher seroprevalence, but this connection was not retained in multivariable models and could be a result of nationality-related confusion. In a broader global context, the resurgence of vaccine-preventable infectious diseases should be interpreted alongside the rapid recovery and expansion of international mobility after the COVID-19 pandemic. Cross-border movement for education, employment, tourism, and economic activity can increase the probability of importing infectious diseases into countries that have already achieved elimination or very low endemic transmission [42]. Mobile populations, including international students and migrant workers, may have heterogeneous vaccination histories, incomplete documentation, and different levels of access to preventive health services. In addition, congregate settings such as dormitories, classrooms, workplaces, and shared accommodations can facilitate secondary transmission once an imported case occurs. Therefore, measles prevention in elimination settings should not rely solely on high national vaccination coverage, but should also address immunity gaps in specific mobile subpopulations.

Most reported cases are associated with importation and subsequent local transmission in nations with eliminated measles. Populations with considerable international mobility, such as international students, may contribute to virus introduction and spread. The significant variation by nationality found in this study underscores the existence of immunity gaps within these migratory populations. Even in nations with high overall vaccination coverage, variation in immunity among subpopulations can jeopardize elimination attempts. An outbreak of measles was reported among healthcare workers in South Korea, which reported 88.9% seropositivity in 2018 [43]. In contrast, international students, acting as the more sensitive population for measles (79.6% seropositivity), will benefit from policy intervention.

The inconsistency between self-reported vaccination history and serological immunity hinders the identification of vulnerable people using traditional screening approaches. In this setting, targeted serological screening and catch-up vaccination programs for high-risk groups, such as international students, may be a more effective and evidence-based strategy. Given the growing scope of global migration, combining targeted surveillance with vaccination measures may be important to maintaining measles eradication status. It is also essential to promote measles education, pre-semester vaccination programs, vaccination coverage, and vaccination registries [41,44,45,46,47]. Thus, these findings suggest the need for proactive and integrated preparedness measures for epidemic threats on campus from a health policy perspective. We propose the following comprehensive strategies, spanning infectious disease prevention and risk early warning. Firstly, a serological screening upon university admission or verification of vaccination records may be beneficial for all students, both domestic and foreign, especially those with unclear or undocumented immunization histories. Universities should establish entry-based immunization verification systems as the first defense to prevent infections. Secondly, ‘catch-up’ vaccination programs should be operated before or during the early semester as the second defense for students with seronegative results. Meanwhile, multilingual education materials should be offered to improve accessibility and acceptance. Third, rapid reporting and response protocols should be established for suspected measles cases in dormitories and other congregate settings, including immediate isolation, contact tracing, post-exposure prophylaxis, and temporary risk communication. Finally, national immunization policy should consider mobile populations as a priority group for targeted surveillance, rather than relying only on aggregate national vaccination coverage. These measures could be incorporated into university health systems and regional infectious disease preparedness plans, creating a coordinated framework among universities, local governments, public health centers, and national disease control authorities.

This study has several limitations that should be considered. First, the causal association between the observed parameters and seropositivity cannot be established due to the cross-sectional design. Second, participants were recruited from two universities in a single province, which may limit the generalizability and introduce potential selection bias, as participants may differ from non-participants in health awareness or vaccination behavior. Third, vaccination history, previous measles infection, and overseas travel history were self-reported and cannot be verified using official records, which may introduce recall, response, and social desirability biases, and response bias. In addition, non-response bias cannot be excluded due to the difference between participants and non-participants. However, multilingual questionnaires, standardized survey procedures, and serological confirmation were used to reduce potential information bias. Therefore, self-reported vaccination-related variables should be interpreted cautiously and should not be considered equivalent to confirmed vaccination coverage. Finally, the ELISA results cannot distinguish vaccine-induced immunity from immunity acquired through natural infection, although they provide an objective measure of measles IgG positivity. Thus, the findings reflect overall population immunity rather than direct vaccination coverage. Although the absence of a local comparison group may limit the direct contextual comparison of the results, the analysis of immune variability across various epidemiological backgrounds is made possible by the participation of students from major source countries. Future studies that include avidity testing, which can assist in distinguishing recent infection from long-term immunity (whether vaccine-induced or natural), would be important in determining the precise origins of immunity and adapting public health messages accordingly.

## 5. Conclusions

This cross-sectional serological study found that measles IgG seropositivity among international students was 79.6%, substantially below the 95% threshold generally required for herd immunity. Seropositivity differed markedly by nationality, with Central Asian students showing the lowest level of immunity, while self-reported vaccination history was not significantly associated with serological immunity. These findings indicate that questionnaire-based screening alone may be insufficient to identify susceptible students. University-based immunization record verification, targeted serological screening, and catch-up vaccination should be considered as part of preparedness strategies for preventing importation-related measles transmission in highly mobile student populations.

## Figures and Tables

**Table 1:** Baseline characteristics among international student participants based on self-reported data (*n* = 299).

Category		*n*	%
Nationality	Central Asia	95	31.8
	Vietnam	75	25.1
	China ^a^	129	43.1
Sex	Male	173	57.9
	Female	126	42.1
Age group (years)	17–20	121	40.5
	21–30	156	52.2
	≥31	22	7.3
Date of Entry to Korea	Before 2024	31	10.4
	February–June 2024	45	15.1
	July–October 2024	221	73.9
Type of Academic Program	Language institute	137	45.8
	Undergraduate	58	19.4
	Graduate	104	34.8
Current Health Condition	Yes	3	1
	No	290	97
Present Medication Use	Yes	5	1.7
	No	289	96.7
MCV Status ^b^	Yes	75	25.1
	No	157	52.5
	Unknown	67	22.4
MCV Dose	1 dose	31	41.3
	≥2 doses	44	58.6
History of Measles Infection	Yes	4	1.3
	No	269	90
	Unknown	25	8.4
Current Residence Type	Dormitory	226	75.6
	Private rental	73	24.4

^a^ China: The People’s Republic of China includes Hong Kong, Macao, and Taiwan. ^b^ MCV: measles-containing vaccine; statistical analysis: Pearson’s chi-square test.

**Table 2:** Factors associated with measles IgG seropositivity among international students, overall and among participants aged ≤30 years. (Missing responses were excluded from the analysis for individual variables; therefore, denominators may vary across categories and may be smaller than the total sample size (*n* = 299) or the subgroup aged ≤30 years (*n* = 277). Percentages were calculated using available-case analysis).

Variables	Category	Participants (*n* = 299)			Participants Aged ≤30 Years (*n* = 277)		
		Seroprevalence (*n*, %)	95% CI	*p*-Value	Seroprevalence (*n*, %)	95% CI	*p*-Value
Overall	–	238 (79.6)	74.7–83.9	–	216 (78.0)	72.8–82.5	–
Sex *	Male	127 (73.4)	66.4–79.6	0.002	116 (71.6)	63.2–78.9	0.002
	Female	111 (88.1)	81.3–93.1		100 (86.9)	79.2–92.3	
Age group (years) *	17–20	85 (70.2)	61.5–78.1	0.001	85 (70.2)	60.2–78.8	0.006
	21–30	131 (84.0)	77.5–89.2		131 (84.0)	76.9–89.5	
	≥31	22 (100.0)	84.6–100	-	-	-	
Nationality **	China	122 (94.6)	89.3–97.8	<0.001	100 (93.4)	86.8–97.1	<0.001
	Vietnam	61 (81.3)	70.9–89.3		61 (81.3)	70.0–89.3	
	Central Asia	55 (57.9)	47.5–67.8		55 (57.9)	44.9–70.0	
Period of Entry to Korea	Before 2024	26 (83.9)	66.3–94.5	0.83	22 (73.3)	52.9–87.2	0.52
	February–June 2024	36 (80.0)	65.4–90.4		36 (86.7)	72.8–94.2	
	July–October 2024	175 (79.2)	73.3–84.3		153 (76.5)	69.4–82.5	
Education level **	Language	98 (71.5)	63.3–78.9	<0.001	98 (71.5)	62.2–79.4	<0.001
	Undergraduate	42 (72.4)	59.1–83.3		39 (70.9)	55.2–83.0	
	Graduate	98 (94.2)	87.8–97.8		79 (92.9)	85.2–96.8	

Statistical analysis: Pearson’s chi-square test, Kruskal–Wallis chi-squared test, or Fisher’s exact test. *: *p* < 0.05, **: *p* < 0.001. -: Not applicable.

**Table 3:** Association between self-reported indicators and IgG seropositivity, overall and among participants aged ≤30 years. (Missing or ‘Unknown’ responses were excluded from the variable-specific analyses. Therefore, denominators may differ across variables, and percentages were calculated based only on available valid responses).

Category	Category	Participants (*n* = 299)			Participants Aged ≤30 Years (*n* = 277)		
		Seroprevalence, *n* (%)	95% CI	*p*-Value	Seroprevalence, *n* (%)	95% CI	*p*-Value
Currently Health Condition	Yes	3 (100.0)	29.2–100.0	1	3 (75.0)	22.1–98.7	0.63
	No	232 (80.0)	74.9–84.5		213 (78.0)	71.8–83.4	
Currently on medication	Yes	4 (80.0)	28.4–99.5	1	3 (75.0)	64.9–86.5	0.63
	No	231 (79.9)	74.8–84.4		213 (78.0)	71.8–83.4	
MCV ^1^ Status	Yes	60 (76.9)	66.0–85.7	0.10	58 (77.3)	64.9–86.5	0.34
	No	135 (86.0)	79.5–91.0		113 (83.7)	75.7–89.7	
Number of MCV Doses	1 dose	25 (75.8)	57.7–88.9	0.53	25 (75.7)	56.3–88.7	0.53
	≥2 doses	20 (83.3)	62.6–95.3		20 (83.3)	78.9–87.7	
History of Measles Infection	Yes	4 (100.0)	39.8–100.0	0.58	4 (100)	51.0–100	0.28
	No	211 (78.4)	73.1–83.1		189 (76.5)	70.0–82.0	
Current Residence Type	Dormitory	175 (77.4)	71.5–82.7	0.10	161 (75.9)	68.7–81.9	0.19
	Private rental	63 (86.3)	76.3–93.2		55 (84.6)	72.8–92.0	
Overseas Travel History **	Yes	90 (93.8)	86.8–97.7	<0.001	71 (92.2)	83.8–96.6	<0.001
	No	145 (72.5)	65.7–78.6		145 (72.5)	64.7–79.3	

Statistical analysis: Fisher’s exact test. ^1^: measles-containing vaccine; **: *p* < 0.001.

**Table 4:** Logistic regression for sustained associated factors, overall and among participants aged ≤30 years.

Variables	Categories	Participants (*n* = 299)			Participants Aged ≤30 Years (*n* = 277)		
		Adjusted OR	95% CI	*p*-Value	Adjusted OR	95% CI	*p*-Value
Sex	Male	-	-	-	-	-	Ref
	Female	1.40	0.66–2.98	0.38	1.41	0.66–3.07	0.85
Age group (years)	17–20 years	0.49	0.003–5.3	0.61	-	-	Ref
	21–30 years	0.43	0.003–4.10	0.54	0.90	0.44–1.87	0.85
	≥30	-	-	Ref	-	-	-
Nationality	Central Asia	-	-	-	-	-	Ref
	Vietnam *	2.34	1.05–5.44	0.038	2.43	1.07–5.77	0.006
	China **	7.36	2.42–24.16	<0.001	8.35	2.67–28.50	<0.001
Education level	Language institute	-	-	-	-	-	Ref
	Undergraduate	0.45	0.18–1.09		0.44	0.17–1.09	0.08
	Graduate	1.11	0.36–3.59	0.08	1.13	0.36–3.85	0.84
Overseas travel	Yes	-	-	-	-	-	Ref
	No	0.52	0.18–1.37	0.27	0.51	0.16–1.38	0.20

Statistics analysis: multivariate logistic regression. * *p* < 0.05; ** *p* < 0.001. -: Not applicable.

## Data Availability

The data used in this work are available from the corresponding author upon reasonable request.

## Abbreviations

The following abbreviations are used in this manuscript:

IgG	Immunoglobulin G
ELISA	enzyme-linked immunosorbent assay
COVID-19	coronavirus disease
CSF	cerebrospinal fluid
TMB	3,3′,5,5′-tetramethylbenzidine
EPI	Expanded Program on Immunization
SIA	Supplementary Immunization Activity
MCV	measles-containing vaccine
